# A 21-day Daniel fast with or without krill oil supplementation improves anthropometric parameters and the cardiometabolic profile in men and women

**DOI:** 10.1186/1743-7075-9-82

**Published:** 2012-09-13

**Authors:** John F Trepanowski, Mohammad M Kabir, Rick J Alleman, Richard J Bloomer

**Affiliations:** 1 Cardiorespiratory/Metabolic Laboratory, 161 Roane Field House, University of Memphis, Memphis, TN, 38152, USA

**Keywords:** Vegan, Blood lipids, Blood glucose, Blood pressure

## Abstract

**Background:**

The Daniel Fast is a vegan diet that prohibits the consumption of animal products, refined foods, white flour, preservatives, additives, sweeteners, flavorings, caffeine, and alcohol. Following this dietary plan for 21 days has been demonstrated to improve blood pressure, LDL-C, and certain markers of oxidative stress, but it has also been shown to lower HDL-C. Krill oil supplementation has been shown to increase HDL-C.

**Methods:**

We investigated the effects of following a Daniel Fast dietary plan with either krill oil supplementation (2 g/day) or placebo supplementation (coconut oil; 2 g/day) for 21 days. The subjects in this study (12 men and 27 women) were heterogeneous with respect to body mass index (BMI) (normal weight, overweight, and obese), blood lipids (normolipidemic and hyperlipidemic), blood glucose (normal fasting glucose, impaired fasting glucose, and type 2 diabetic), and blood pressure (normotensive and hypertensive).

**Results:**

Krill oil supplementation had no effect on any outcome measure (all *p* > 0.05), and so the data from the krill oil group and the placebo group were collapsed and analyzed to examine the effects of following a 21-day Daniel Fast. Significant reductions were observed in LDL-C (100.6 ± 4.3 mg/dL vs. 80.0 ± 3.7 mg/dL), the LDL:HDL ratio (2.0 ± 0.1 vs. 1.7 ± 0.1), fasting blood glucose (101.4 ± 7.5 mg/dL vs. 91.7 ± 3.4 mg/dL), fasting blood insulin (7.92 ± 0.80 μU/mL vs. 5.76 ± 0.59 μU/mL), homeostasis model assessment of insulin resistance (HOMA-IR) (2.06 ± 0.30 vs. 1.40 ± 0.21), systolic BP (110.7 ± 2.2 mm Hg vs. 105.5 ± 1.7 mm Hg), and body weight (74.1 ± 2.4 kg vs. 71.5 ± 2.3 kg) (all *p* < 0.05).

**Conclusion:**

Following a Daniel Fast dietary plan improves a variety of cardiometabolic parameters in a wide range of individuals in as little as 21 days, and these improvements are unaffected by krill oil supplementation.

**Trial registration:**

Clinicaltrial.govNCT01378767

## Background

Type 2 diabetes and cardiovascular disease are two epidemics that will grow more prevalent in the near future [1,2]. Dietary treatments that target the main parameters associated with type 2 diabetes and cardiovascular disease – particularly glycemic control, blood lipids, blood pressure, and body weight – will likely reduce the morbidity and mortality associated with these diseases. Vegan diets have been shown to beneficially modulate these parameters [3-5].

A recently investigated “Daniel Fast” (a plant-based dietary plan that prohibits the intake of animal products, refined foods, white flour, preservatives, additives, sweeteners, flavorings, caffeine, and alcohol) was shown to lower blood pressure, total cholesterol, and low density lipoprotein cholesterol (LDL-C) [6]. Other improvements associated with this dietary plan include reductions in malondialdehyde and hydrogen peroxide, as well as increases in the Trolox Equivalent Antioxidant Capacity and nitrate and nitrite (NOx) [6,7].

The Daniel Fast has also been shown to lower high density lipoprotein cholesterol (HDL-C), although the reduction in this cholesterol was outpaced by a greater reduction in LDL-C [6]. Nonetheless, the Daniel Fast would likely confer even greater cardiovascular benefit if HDL-C were able to be maintained. A previous study reported a 43-60% increase in HDL-C in human volunteers supplementing with krill oil [8]. Based on those findings, the purpose of the present investigation was to compare the effects of krill oil versus placebo supplementation in individuals following a Daniel Fast dietary plan for 21 days. Nineteen of the 39 subjects who completed the Daniel Fast supplemented daily with krill oil, while the remaining 20 subjects supplemented daily with a placebo. However, krill oil supplementation did not affect any primary or secondary outcome measure. Consequently, we pooled together the data for subjects in the krill oil and placebo conditions, and we present the results of following a Daniel Fast dietary plan for 21 days.

## Methods

### Subjects and screening

Subjects were recruited from the Memphis area through the use of flyers placed on and around the University of Memphis campus. Eligibility was assessed via questionnaire. Our prior investigation of a 21-day Daniel Fast indicated that changes in outcome measures in response to the dietary plan were unaffected by gender, baseline body weight, or baseline exercise habits [6]. Therefore, we elected to not place any restrictions on these variables regarding enrollment into the present study. Key inclusion criteria were men and women between the ages of 19–65 years with a body mass index (BMI) between 18–37 kg/m^2^. Key exclusion criteria were smoking, contraindication to krill oil or coconut oil (placebo) supplementation, pregnancy or desire to become pregnant, and unwillingness to follow a vegan diet. As these exclusion criteria were included in the recruitment flyers, outside of self-exclusion, none of the individuals that expressed interest in the study had to be excluded from participating. Sample size for this study was calculated based on the changes in total cholesterol and LDL-C that were observed in our previous investigation of a 21-day Daniel Fast [6]. Forty-one subjects (13 men and 28 women) were enrolled, but 2 subjects (1 man and 1 woman) withdrew during the intervention phase due to personal reasons; therefore, 39 subjects were included in the analysis. Twenty-two subjects were classified as normal weight (BMI < 25 kg/m^2^), 9 were classified as overweight (BMI 25–29.9 kg/m^2^), and 8 were classified as obese (BMI ≥ 30 kg/m^2^). Thirty-three subjects were classified as exercise-trained, which was defined as performing ≥ 2 h/week of combined anaerobic and aerobic exercise of moderate to high intensity. On average, subjects performed 2.1 ± 0.2 h/week of anaerobic exercise for 6.8 ± 1.1 years and 4.7 ± 0.4 h/week of aerobic exercise for 8.3 ± 1.0 years. Six subjects had elevated BP (≥ 140/90 mm Hg), 7 subjects had elevated total cholesterol (≥ 200 mg/dL), and 2 subjects were type 2 diabetics. The following medication usages likely affected some of the outcome measurements in this study: Two subjects were taking hormone replacements, 2 were taking hypothyroid medication, 1 was taking an anti-inflammatory, 1 was taking insulin, 1 was taking hyperthyroid medication, and 1 was taking a stimulant/appetite suppressant. Failure to exclude individuals taking these medications during the screening process can be considered a limitation of this study. Subjects were instructed to not consume antioxidant supplements from 2 weeks prior to beginning the dietary plan until end of study. Prior to participation, each subject was informed of all procedures, potential risks, and benefits associated with the study in both verbal and written form in accordance with the approved procedures of the University Institutional Review Board for Human Subjects Research (protocol H11-14). Subjects provided written informed consent prior to being enrolled in the study.

The dietary intervention consisted of only consuming foods that comply with a Daniel Fast for 21 days. The 21-day duration was chosen to maximize generalizability – when motivated by religious reasons, most individuals follow a Daniel Fast dietary plan for 21 days, although 10- and 40-day Daniel Fasts have also been observed – and also to allow for a comparison against the findings from our previous study of the Daniel Fast [6]. No meals were provided, and energy intake was unrestricted. Subjects were permitted to consume any food or beverage so long as it contained no animal products, white flour, preservatives, additives, sweeteners, flavorings, caffeine, or alcohol. Special emphasis was made to instruct the subjects to check the ingredients label of every food or beverage they planned to consume to ensure that it completely lacked any of the proscribed ingredients mentioned above. As an additional precaution, subjects were provided a list of commonly consumed foods that comply with the Daniel Fast as well as a second list of commonly consumed foods that do not. Subjects were also provided a basic recipe guide that only incorporated ingredients that comply with the Daniel Fast.

The outcome variables described below were measured immediately before (day 1) and after following the Daniel Fast dietary plan (day 22). All data collection occurred between 05:00 and 11:00 hours while subjects were in a 12-hour post-absorptive state.

### Supplementation

Subjects were randomly assigned to consume one of the following for the duration of the Daniel Fast: Twenty-one subjects (7 men and 14 women) were assigned to orally consume krill oil capsules (NOW Foods, Bloomingdale, IL: 2 g/day in 2 daily dosages of 1 g), and 20 subjects (7 men and 13 women) were assigned to orally consume placebo capsules (coconut oil; NOW Foods, Bloomingdale, IL: 2 g/day in 2 daily dosages of 1 g). Specifically, men and women were alternately assigned to the krill oil group and the placebo group as they were enrolled in the study. Both the subjects and the investigators were blind to treatment via the use of coded bottles. A recent investigation examining the safety and tolerability of krill oil supplementation at 2 g/day for 4 weeks indicated that “krill oil was generally well tolerated and did not show evidence of any adverse influence on safety parameters” [9]. Both of the subjects that withdrew from the study were in the krill oil group; thus, 19 subjects (6 men and 13 women) in the krill oil group completed the intervention. Subjects were instructed to consume 1 capsule in the morning with breakfast and another capsule in the evening with dinner, daily. Capsule counts upon bottle return at the end of the intervention were used to determine compliance to the supplementation. Each capsule of krill oil provided 230–300 mg of Omega-3 fatty acids, 140–160 mg of EPA, 80–90 mg of DHA, 10–20 mg of Omega-6 fatty acids, 80–90 mg of Omega-9 fatty acids, 390–420 mg of phospholipids, and 1.0-1.5 mg of esterified astaxanthin.

### Blood pressure and heart rate

Subjects were seated in a chair with a BP cuff placed on their left arm. After a 10-minute period of quiet rest, two technicians measured heart rate by palpating the radial artery for 60 seconds. Blood pressure was then measured via auscultation using a dual-earpiece stethoscope that allowed for two technicians to listen simultaneously. Duplicate measures were obtained for both heart rate and BP, and the average of all measures was used in data analysis. If values deviated by more than 5 beats/minute for heart rate or 5 mm Hg for BP, an additional measure was taken.

### Anthropometric variables

Subjects’ height was measured using a stadiometer, and bodyweight was measured using a calibrated medical scale. Waist and hip circumference measurements were obtained using a tension-regulated measuring tape; subjects wore athletic shorts for these measurements. Fat mass, fat-free mass, total body fat percentage, and trunk-specific body fat percentage were determined via dual energy x-ray absorptiometry (Hologic QDR-4500 W) using a 4-min fan array.

### Blood collection and biochemical variables

Blood samples that were collected in tubes containing EDTA were immediately separated via centrifugation at 1500 g for 15 minutes at 4°C for collection of plasma. Blood samples that were collected in tubes containing no additives were allowed to clot at room temperature for 30 minutes and then separated by centrifugation at 1500 g for 15 minutes at 4°C for collection of serum. A portion of blood samples were analyzed within 1 day of collection as follows: A complete blood count was performed using an automated cell counter (Coulter LH750). A comprehensive metabolic panel was obtained using automated procedures (Roche/Hitachi Modular). A lipid panel was obtained using enzymatic procedures (Roche/Hitachi Modular). C-reactive protein was measured using a high-sensitivity, particle-enhanced turbidimetric immunoassay (Roche Integra 800). Insulin was measured using an immuno-chemiluminescent assay procedure (Roche Modular E170). The homeostasis model assessment of insulin resistance (HOMA-IR) was calculated as: fasting glucose (mg/dL) × fasting insulin (μU/mL)/405 [10].

The remaining blood samples were immediately stored in multiple aliquots at −70°C until analysis for the following: Malondialdehyde was analyzed in plasma following the procedures of Jentzsch et al. [11] using reagents purchased from Northwest Life Science Specialties (Vancouver, WA). Hydrogen peroxide was analyzed in plasma using the Amplex Red reagent method as described by the manufacturer (Molecular Probes, Invitrogen Detection Technologies, Eugene, OR). NOx was analyzed in plasma using a commercially available colorimetric assay kit (Cayman Chemical, Ann Arbor, MI) according to the procedures provided by the manufacturer. Antioxidant capacity was analyzed in serum using the Trolox Equivalent Antioxidant Capacity assay according to the procedures outlined by the reagent provider (Sigma Chemical, St. Louis, MO). Resistin and adiponectin were analyzed in serum using an enzyme immunoassay according to the procedures provided by the manufacturer (SPI-Bio, Berin Pharma, France).

### Dietary records and physical activity

All subjects were instructed to maintain their normal diet until they began the Daniel Fast. During the 7 days immediately prior to beginning the Daniel Fast, subjects recorded all food and beverages consumed. Subjects also recorded all food and beverages consumed during the final 7 days of the Daniel Fast. These dietary records were analyzed using Food Processor SQL, version 9.9 (ESHA Research, Salem, OR). Subjects were instructed to maintain their normal physical activity and exercise habits during the entire study period with one exception: Subjects were instructed to refrain from strenuous exercise during the 48 hours immediately preceding the 2 assessment days. Subjects were also instructed to refrain from alcohol consumption during the Daniel Fast (as detailed in the dietary guidelines) and also during the 48 hours that preceded day 1.

### Compliance to food intake, rating of physical and mental health, and satiety

At the conclusion of the intervention period (day 22), subjects completed a questionnaire pertaining to their experience with the Daniel Fast. Using a scale of 1–10 (1 = as low as possible; 10 = as high as possible), subjects rated their feeling of physical health and vitality, their mental health (overall outlook and mood), and their level of satiety while following the dietary plan. Subjects also rated their compliance to the Daniel Fast using a scale of 0–100 (0 = complete noncompliance; 100 = complete compliance).

### Statistical analysis

All outcome measures were analyzed using a 2 (condition) × 2 (pre/post intervention) ANOVA. However, data for both conditions were subsequently collapsed and analyzed using a paired t-test. Analyses were performed using JMP statistical software (version 4.0.3, SAS Institute, Cary, NC). Statistical significance was set at *p* ≤ 0.05.

## Results

Thirty-nine subjects successfully completed the study. Compliances to the krill oil and placebo supplementations were 92.4 ± 1.7% and 92.8 ± 2.2%, respectively. No condition (krill oil or placebo) or interaction effects were noted for any primary or secondary outcome measure (*p* > 0.05). Based on these analyses, data for all variables were collapsed, analyzed using a paired t-test, and are presented as pre and post intervention.

Subjects’ self-reported compliance to the dietary plan was 97.8 ± 0.4%. Subjects’ ratings of physical health and vitality, mental health, and level of satiety during the Daniel Fast were 8.3 ± 0.2, 8.6 ± 0.2, and 8.0 ± 0.2, respectively.

Blood pressure and anthropometric data are presented in Table 1. Systolic BP decreased (110.7 ± 2.2 mm Hg vs. 105.5 ± 1.7 mm Hg; *p* = 0.04), while the decrease in diastolic BP was not statistically significant (71.5 ± 2.3 mm Hg vs. 66.9 ± 1.8 mm Hg; *p* = 0.11). Body weight (74.1 ± 2.4 kg vs. 71.5 ± 2.3 kg), fat mass (21.9 ± 1.5 kg vs. 20.8 ± 1.5 kg), and fat-free mass (52.2 ± 2.0 kg vs. 50.8 ± 1.9) each decreased (all *p* < 0.01).

**Table 1:** Blood pressure and anthropometric data before and after a 21-day Daniel Fast (Mean values and standard error of mean (SEM), n = 39)

**Variable**	**Pre**	**Post**	***p*value**^a^
Heart Rate (beats/minute)	66.5 ± 1.6	66.5 ± 1.8	0.82
Systolic Blood Pressure (mm Hg)	110.7 ± 2.2	105.5 ± 1.7	0.04
Diastolic Blood Pressure (mm Hg)	71.5 ± 2.3	66.9 ± 1.8	0.11
Body Weight (kg)	74.1 ± 2.4	71.5 ± 2.3	< 0.01
BMI (kg/m^2^)	25.9 ± 0.7	25.0 ± 0.7	< 0.01
Waist (cm)	86.8 ± 2.0	85.5 ± 2.0	< 0.01
Hip (cm)	101.2 ± 1.8	100.4 ± 1.7	0.01
Waist:Hip	0.85 ± 0.01	0.86 ± 0.01	0.01
Total Body Fat (%)	29.4 ± 1.5	28.8 ± 1.6	0.02
Trunk Body Fat (%)	28.4 ± 1.7	27.3 ± 1.8	< 0.01
Fat Mass (kg)	21.9 ± 1.5	20.8 ± 1.5	< 0.01
Fat-Free Mass (kg)	52.2 ± 2.0	50.8 ± 1.9	< 0.01

^a^*p* values represent the results of paired t-tests.

Cardiometabolic-, oxidative stress-, and inflammatory data are presented in Table 2. LDL-C (100.6 ± 4.3 mg/dL vs. 80.0 ± 3.7 mg/dL), HDL-C (56.0 ± 2.6 mg/dL vs. 50.9 ± 2.6 mg/dL) and the LDL:HDL ratio (2.0 ± 0.1 vs. 1.7 ± 0.1) each decreased (all *p* < 0.01). No changes were observed in serum triglycerides or VLDL (both *p* > 0.05). Blood glucose (101.4 ± 7.5 mg/dL vs. 91.7 ± 3.4 mg/dL), blood insulin (7.92 ± 0.80 mg/dL vs. 5.76 ± 0.59 mg/dL), and HOMA-IR (2.06 ± 0.30 vs. 1.40 ± 0.21) each decreased (all *p* < 0.05). Resistin (6.06 ± 0.31 ng/mL vs. 6.72 ± 0.32 ng/mL) and NOx (19.26 ± 2.56 μmol/L vs. 28.49 ± 3.53 μmol/L) increased (both *p* < 0.05). No changes were observed in adiponectin, malondialdehyde, hydrogen peroxide, Trolox Equivalent Antioxidant Capacity, or C-reactive protein (all *p* > 0.05).

**Table 2:** Cardiometabolic-, oxidative stress-, and inflammatory data before and after a 21-day Daniel Fast (Mean values and standard error of mean (SEM), n = 39)

**Variable**	**Pre**	**Post**	***p*value**^a^
Total Cholesterol (mg/dL)	173.1 ± 5.0	146.0 ± 4.8	< 0.01
LDL-C (mg/dL)	100.6 ± 4.3	80.0 ± 3.7	< 0.01
HDL-C (mg/dL)	56.0 ± 2.6	50.9 ± 2.6	< 0.01
Triglycerides (mg/dL)	82.5 ± 7.4	75.2 ± 5.7	0.18
VLDL (mg/dL)	16.5 ± 1.5	15.1 ± 1.1	0.20
LDL:HDL	2.0 ± 0.1	1.7 ± 0.1	< 0.01
Total:HDL	3.3 ± 0.2	3.1 ± 0.1	< 0.01
Glucose (mg/dL)	101.4 ± 7.5	91.7 ± 3.4	0.04
Insulin (μU/mL)	7.92 ± 0.80	5.76 ± 0.59	< 0.01
HOMA-IR	2.06 ± 0.30	1.40 ± 0.21	< 0.01
Resistin (ng/mL)	6.06 ± 0.31	6.72 ± 0.32	< 0.01
Adiponectin (μg/mL)	16.11 ± 0.88	16.49 ± 0.86	0.22
Malondialdehyde (μmol/L)	0.73 ± 0.06	0.71 ± 0.07	0.81
Hydrogen Peroxide (μmol/L)	4.66 ± 0.37	4.47 ± 0.38	0.57
NOx (μmol/L)	19.26 ± 2.56	28.49 ± 3.53	0.01
TEAC (mmol/L)	0.66 ± 0.01	0.62 ± 0.02	0.56
C-Reactive Protein (ng/mL)	1.90 ± 0.60	1.36 ± 0.39	0.14

HOMA-IR, homeostasis model assessment of insulin resistance; NOx, nitrate and nitrite; TEAC, Trolox Equivalent Antioxidant Capacity.

^a^*p* values represent the results of paired t-tests.

Dietary data are presented in Table 3. Energy intake decreased (1857.6 ± 94.4 kcal/day vs. 1601.7 ± 84.7 kcal/day), as did intake of total fat (66.6 ± 3.8 g/day vs. 54.9 ± 4.7 g/day), saturated fat (21.0 ± 1.3 g/day vs. 8.4 ± 0.8 g/day), trans fat (0.9 ± 0.2 g/day vs. 0.2 ± 0.1 g/day), and dietary cholesterol (224.4 ± 21.0 mg/day vs. 12.2 ± 5.1 mg/day) (all *p* < 0.05). Fiber intake increased (20.7 ± 1.7 g/day vs. 40.0 ± 2.5 g/day; *p* < 0.01).

**Table 3:** Dietary data before and during the final 7 days of a 21-day Daniel Fast (Mean values and standard error of mean (SEM), n = 39)

**Variable**	**Pre**	**Post**	***p*value**^a^
Energy (kcal)	1857.6 ± 94.4	1601.7 ± 84.7	< 0.01
Protein (g)	79.4 ± 6.2	53.0 ± 3.4	< 0.01
Protein (% energy)	16.7 ± 0.7	13.2 ± 0.5	< 0.01
Carbohydrate (g)	229.2 ± 12.5	240.5 ± 14.1	0.35
Carbohydrate (% energy)	49.5 ± 1.0	60.7 ± 1.7	< 0.01
Fiber (g)	20.7 ± 1.7	40.0 ± 2.5	< 0.01
Sugar (g)	81.6 ± 5.7	73.2 ± 5.2	0.15
Fat (g)	66.6 ± 3.8	54.9 ± 4.7	0.02
Fat (% energy)	32.2 ± 0.8	30.1 ± 1.7	0.25
Saturated Fat (g)	21.0 ± 1.3	8.4 ± 0.8	< 0.01
Monounsaturated Fat (g)	13.7 ± 1.6	18.3 ± 2.7	0.07
Polyunsaturated Fat (g)	6.7 ± 0.5	9.2 ± 1.0	0.01
Trans Fat (g)	0.9 ± 0.2	0.2 ± 0.1	< 0.01
Omega-3 (mg)	0.5 ± 0.1	0.6 ± 0.1	0.81
Omega-6 (mg)	4.8 ± 0.4	6.0 ± 0.9	0.13
Dietary Cholesterol (mg)	224.4 ± 21.0	12.2 ± 5.1	< 0.01
Vitamin C (mg)	64.5 ± 5.6	140.6 ± 11.1	< 0.01
Vitamin E (mg)	5.7 ± 0.8	7.7 ± 0.8	0.03
Vitamin A (RE)	362.0 ± 42.4	485.5 ± 52.1	0.02
Selenium (μg)	46.9 ± 4.9	38.4 ± 9.1	0.35

^a^*p* values represent the results of paired t-tests.

## Discussion

### Krill oil supplementation

We sought to determine if krill oil supplementation could maintain HDL-C in individuals following a Daniel Fast dietary plan for 21 days. This was not the case, as HDL-C decreased similarly regardless of whether krill oil or a placebo was consumed. Krill oil supplementation also did not affect any other outcome measure. Our findings therefore strongly contradict the work of Bunea et al. [8], who reported that krill oil supplementation (1–3 g/day for 90 days) increased HDL-C (43-60%) and decreased LDL-C (32-39%). On the other hand, our findings support the results of recent investigations [9,12,13] that found no effect for krill oil supplementation (2 g/day for 28 days, 1 g/day for 42 days, and 3 g/day for 49 days, respectively) on blood lipids. The baseline LDL-C values in the Bunea et al. study (165–183 mg/dL) [8] were considerably larger than the corresponding values of the other investigations mentioned in this paragraph (including the present study) [9,12,13]. This may explain why only Bunea and colleagues [8] found that krill oil supplementation reduced LDL-C. However, baseline HDL-C concentrations were similar across studies (also including the present study) [8,9,12,13], leaving little scientific explanation for the striking improvement in this cholesterol noted by Bunea et al. [8]. Due to the inability of krill oil supplementation to affect any outcome measure in the present study, values for subjects in both the krill oil and placebo conditions were collapsed and are discussed as such from this point forward.

### Collapsed data

Following a Daniel Fast dietary plan resulted in clinically significant reductions in LDL-C, the LDL:HDL ratio, blood glucose, blood insulin, HOMA-IR, systolic BP, and body weight. This study demonstrates that meaningful improvements to cardiovascular and glycemic parameters can be obtained in response to dietary changes in only 21 days.

Plant-based diets have consistently been shown to reduce LDL-C [14]. The main dietary changes responsible for this are likely reductions in total fat, saturated fat, and dietary cholesterol intake, as well as an increase in fiber intake [14]; each of these dietary changes was observed in this study. HDL-C decreased in response to the dietary plan, and decreases in this cholesterol have been observed in other studies examining vegan diets [3,6]. A recent investigation found that substituting monounsaturated fat for carbohydrate within a vegetarian diet increased HDL-C by 12.5% [15]. A similar approach should be considered for future investigations of the Daniel Fast.

The reductions in blood glucose, blood insulin, and HOMA-IR in the present study were not observed in our previous investigation of the Daniel Fast [6]. Smaller baseline values for each of the variables in the previous investigation [6] likely explain these discrepancies. A trial examining a vegan diet in nondiabetic, overweight and obese, postmenopausal women noted similar improvements in blood glucose and blood insulin to those of the present investigation [4]. Vegan diets have also been shown to improve glycemic control in individuals with type 2 diabetes [3,5,16].

The reduction in systolic BP may have been mediated by an increase in nitric oxide, a signaling molecule that promotes vasodilation [17]. Support for this claim comes from our observed 45% increase in the surrogate marker for nitric oxide, NOx, which is likely a consequence of increased vegetable consumption (leafy green vegetables in particular) [18].

There were some limitations associated with this study. First, the study lacked a control group of subjects maintaining their usual diet during the 21-day intervention phase. Many prior investigations have demonstrated that our measured outcome variables experience minimal to no change in 21 days in the absence of modification in dietary habits, exercise habits, or medication usage. Nonetheless, such changes are possible, and our failure to include a control group clouds our ability to determine how much of the changes in our measured outcome variables were due to dietary changes. Second, as mentioned above, some subjects were enrolled in the study despite using medications that may have affected outcome measures. Finally, the inclusion criteria were broad, making it difficult to generalize the results of this investigation.

A key strength of this study is that the observed changes were of sizeable magnitudes despite occurring in individuals whose average baseline values for BMI (25.9 kg/m^2^), LDL-C (100.6 mg/dL), blood glucose (101.4 mg/dL), and systolic BP (110.7 mm Hg) did not differ greatly from healthy norms (< 25.0 kg/m^2^, < 100 mg/dL, < 100 mg/dL, and < 120 mm Hg respectively). Insofar as healthy individuals have less “room for improvement”, this suggests that individuals with less-desirable values for these variables could potentially derive greater clinical benefit from this dietary plan. As mentioned above, this was observed when the results of this study were compared to those of our previous investigation of the Daniel Fast [6] (i.e., the greater improvements in glycemic variables noted in this study were likely due to less-healthy baseline values). Therefore, it is important that future trials examining the Daniel Fast include subject populations that would likely experience the greatest clinical benefit from this dietary plan, such as individuals with type 2 diabetes, hyperlipidemia, hypertension, and/or overweight/obesity.

## Conclusion

A Daniel Fast dietary plan improves blood lipids, blood glucose, blood insulin, HOMA-IR, systolic BP, and body weight. These improvements were unaffected by krill oil supplementation, they occurred in only 21 days, and they occurred in a group of subjects who, on average, had less room for improvement compared to many diseased populations. Whether following the dietary plan for a longer duration increases the magnitudes of these improvements, and whether less-healthy individuals respond more favorably to the dietary plan, are both presently unknown. These are exciting topics for future research.

## Competing interests

The krill oil and placebo capsules were a gift from NOW Foods (Bloomingdale, IL). None of the authors have a financial interest in the krill oil used in this study or in NOW Foods. There are no known or perceived conflicts of interest for any author.

## Authors’ contributions

The authors’ contributions were as follows: RJB designed the experiment, performed the laboratory analyses, analyzed the data, assisted with the preparation of the manuscript, and has primary responsibility for the final content. JFT, MMK, and RJA conducted the clinical trial. JFT also wrote the manuscript. All authors read and approved the final manuscript.
